# Ossified Pulp

**Published:** 1872-06

**Authors:** 


*Ossified Pulp.*—Dr. R. D. Murrell, of Chattanooga, Tennessee, sends us the following history of a case in his practice:

"Enclosed please find nerve of second superior molar, from the palatine fang; the nerves in both of the buccal fangs of the tooth were dead. The patient complained of pain in the jaw, cheek under the antrum, and forehead. After a careful diagnosis I pronounced it neuralgia, and treated him in the usual manner, but without any beneficial result. I then examined his teeth, and found that the third molar was diseased under the enamel, whereupon I advised its extraction. This was done, but still no benefit. In a day or two I made another examination, and found that by pressing upon a gold filling in the crown of the second molar it was very sensitive. Inquiring how long the filling had been in the tooth, he informed me that it had been inserted thirteen years before. I removed the filling and found the nerve exposed, to which I applied nerve paste and destroyed its sensitiveness; still no relief. I then advised the immediate extraction of the tooth; and after its removal split the fangs and found the upper end of the nerve dead from the effects of the nerve paste, but the remaining portion in a healthy condition, except the centre, which was occupied with an osseous substance. The patient is still suffering with neuralgia, but it is now confined to his forehead and crown of the head. There is no pain in the jaw now, ten days after the extraction of the tooth."

